# Altered *MCF2L-AS1* expression and correlation with the prognosis of diabetic nephropathy

**DOI:** 10.3389/ebm.2025.10771

**Published:** 2026-01-22

**Authors:** Nastaran Injinari, Morteza Hadizadeh, Nasim Namiranian, Seyed Mehdi Kalantar, Ali Dehghani Firoozabadi, Samira Asadollahi

**Affiliations:** 1 Diabetes Research Center, Non-Communicable Diseases Research Institute, Shahid Sadoughi University of Medical Sciences, Yazd, Iran; 2 Department of Persian Medicine, School of Persian Medicine, Shahid Sadoughi University of Medical Sciences, Ardakan, Yazd, Iran; 3 Physiology Research Center, Institute of Neuropharmacology, Kerman University of Medical Sciences, Kerman, Iran; 4 Department of Genetics, Faculty of Medicine, Shahid Sadoughi University of Medical Sciences, Yazd, Iran; 5 Meybod Genetic Research Center, Meybod, Yazd, Iran; 6 Yazd Cardiovascular Research Center, Non-communicable Disease Research Institute, Shahid Sadoughi University of Medical Sciences, Yazd, Iran; 7 Research Center for Food Hygiene and Safety, Shahid Sadoughi University of Medical Sciences, Yazd, Iran

**Keywords:** diabetic nephropathy, *MCF2L-AS1*, *BCOR*, molecular mechanisms, gene expression

## Abstract

Although diabetic nephropathy (DN) stands as a prominent complication in individuals with diabetes, the specific molecular mechanisms remain unclear. In this study, we focused on one newly discovered lncRNA, *MCF2L-AS1*, and its target gene, *BCOR*, in individuals with various levels of DN. Twenty-eight participants with different stages of DN (14 early stage and 14 late stage), 12 non-diabetic individuals, and 12 with T2DM without microvascular complications were selected. The qPCR was done, and one-way ANOVA assessed gene expression. ROC curves analysis and Spearman correlations between levels of expression and clinicopathological parameters were explored. The expression of *MCF2L-AS1* decreased in the early and late DN groups compared to the type 2 diabetes (T2DM) (P = 0.01 and P = 0.03, respectively) and non-diabetic groups (P = 0.01 and P = 0.03, respectively). However, *BCOR* gene expression analysis revealed that there was no significant difference between the groups (P = 0.27). *MCF2L-AS1* levels negatively correlated with microalbuminuria (P = 0.003, r = −0.41), but not with creatinine (Cr) (P = 0.058, r = −0.29). Moreover, there was no correlation between *BCOR* and microalbumin (P = 0.85, r = 0.02) and Cr (P = 0.49, r = 0.10). ROC curves underscored significant diagnostic accuracy for *MCF2L-AS1* in distinguishing DN from people without kidney diseases (P < 0.05). This study introduces *MCF2L-AS1* as a potential key player in the molecular landscape of DN, shedding light on its multifaceted interactions. The results provide a basis for further exploration and therapeutic interventions in the management of DN.

## Graphical Abstract

**Figure:**
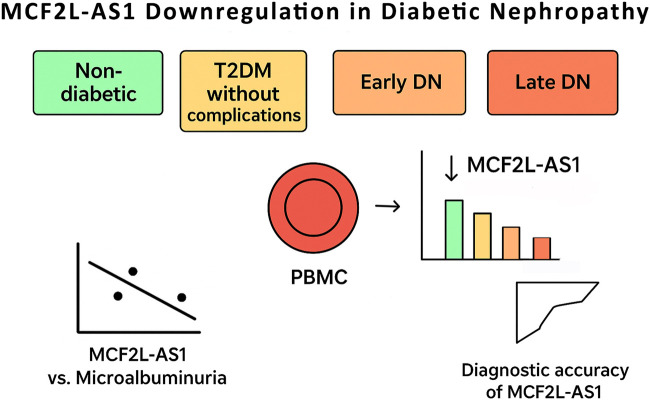

## Impact statement

This study is significant in identifying *MCF2L-AS1*, a long non-coding RNA, as a novel molecular component potentially involved in diabetic nephropathy (DN), a major diabetes complication with unclear mechanisms. Its downregulation in DN patients and correlation with clinical markers like microalbuminuria suggest *MCF2L-AS1* as a disease-associated molecule worth further investigation. These findings provide a foundation for exploring *MCF2L-AS1*’s role in DN pathogenesis and its potential as a biomarker or therapeutic target, thereby contributing to advancing understanding and management of kidney dysfunction in diabetes.

## Introduction

Diabetic nephropathy (DN) poses a significant challenge within the realm of diabetes-related complications [1]. This condition can progress relentlessly toward end-stage renal disease (ESRD), necessitating interventions like dialysis or transplantation [2]. Unfortunately, the early stages of DN frequently go undetected until advanced structural damage becomes evident [1].

DN is typically characterized by progressive albuminuria and a decline in the glomerular filtration rate (GFR) [3, 4]. Although microalbuminuria is traditionally regarded as the first clinical sign of kidney disease, epidemiologic research shows that 25–50% of individuals with diabetic kidney disease and a GFR below 60 mL/min/1.73 m^2^ are normoalbuminuric, a condition increasingly prevalent with advanced age and female gender [5].

To advance new treatment and diagnosis strategies, gaining a deeper understanding of the mechanisms underlying kidney damage and repair is imperative [6]. In the era of advanced medical research, the role of genetic and epigenetic factors in kidney diseases, including DN, has come to the forefront [7, 8].

Long non-coding RNAs (lncRNAs), constituting over 80% of human genomic transcripts, play crucial roles in regulating gene expression, splicing, and chromatin epigenetic modifications [9–11]. Reports suggest that lncRNAs regulate DN occurrence and progression by influencing factors such as inflammation, oxidative stress, and increased renal accumulation of extracellular matrix (ECM) proteins [7].

For instance, LINC00968, overexpressed in diabetic db/db mouse tissue, accelerates the proliferation and fibrosis of diabetic nephropathy through epigenetic repression of p21 by recruiting *EZH2* [12]. Additionally, upregulation of MIAT in DN exhibits an antagonistic effect on modulating mesangial cell proliferation and fibrosis via targeting *miR-147a* [13].

Recent research employing advanced RNA sequencing (RNA-seq) and chromatin immunoprecipitation (ChIP) sequencing techniques has unveiled numerous lncRNAs with altered expression patterns in DN [14, 15]. Our previous study, based on analyzing differentially expressed genes (DEGs), identified downregulation of lncRNA MCF2L antisense RNA 1 (*MCF2L-AS1*) and upregulation of *BCOR* (BCL-6 corepressor) in peripheral blood mononuclear cells (PBMCs) from individuals with varying DN levels [16].

*MCF2L-AS1* has been recently reported as a new molecule. It has been demonstrated as an oncogene that promotes the development of a variety of malignancies, such as colorectal cancer [17], breast cancer [18], and lung cancer [19]. Despite its prominent role in other diseases, its involvement in diabetes and DN remains uncharted territory.

Thus, in this study, we aimed to validate bioinformatics results by assessing the expression of *MCF2L-AS1* and its potential target gene, *BCOR*, in participants with different DN stages, T2DM patients, and non-diabetic individuals. This research strives to identify molecular targets for promising strategies to control kidney disorders in diabetic patients.

## Materials and methods

### Data collection and gene expression analysis

Firstly, the investigation delved into the expression datasets of both lncRNA and mRNA pertaining to DN. The keywords employed for this exploration included “lncRNA,” “mRNA,” “Diabetic nephropathy,” “peripheral blood,” and “*Homo sapiens*” [porgn: txid9606]. These were utilized against the Gene Expression Omnibus (GEO) database^1^. The limma package, found within Bioconductor, facilitated the analysis of gene expression as well as the identification of statistically significant differentially expressed genes (DEGs). This identification was based on the disparity in expression values between normal and diabetic samples. To be classified as DElncRNAs, the lncRNAs necessitated a log2 fold change ≥ |0.5|, whereas DEmRNAs required an adjusted p-value threshold of 0.05. Lastly, the lncRNA chosen for inclusion in this research was *MCF2L-AS1*.

### Interaction assessment between *MCF2L-AS1* and targets

We established a network to explore the interaction between *MCF2L-AS1* and mRNA in order to gain a deeper understanding of their functionality. To identify these interactions, we utilized the RNA Interactome Database (RNAInter). The examination of *MCF2L-AS1* involved categorizing it as a lncRNA and specifying the species as *H. sapiens*. We focused on interactions involving RNA and Protein, utilizing various detection methods encompassing all predictions, and assigned confidence scores ranging from 0 to 1 to each interaction. Through this process, we obtained a comprehensive list of proteins targeted by differentially expressed lncRNAs from the RNAInter database. Subsequently, we compared the collected mRNA (from RNAInter) with differentially expressed mRNAs using the Venny 2.1 tool^2^. The resulting list represents the predicted targets for each differentially expressed lncRNA in this investigation. Finally, we employed Cytoscape (3.10.1) software to construct lncRNA-protein networks.

### Gene ontology (GO) analysis

In this investigation, our objective was to ascertain primary biological pathways and gene ontology (GO), including biological process (BP), molecular function (MF), and cellular component (CC) that are associated with DN through the utilization of a gene expression analysis methodology. To achieve this, we procured the ultimate targets of *MCF2L-AS1* from an expression study on DN and conducted an analysis utilizing RNAInters. Subsequently, we scrutinized the final compiled list employing the Toppgene database. Moreover, we proceeded to depict the outcomes by employing a chord plot generated utilizing the GOplot package.

### Patients and clinical data collection

This cross-sectional study has been approved by the ethics committees of Shahid Sadoughi University of Medical Sciences, Yazd, Iran (IR.SSU.REC.1400.191). Written informed consent was given by all study subjects.

The study was conducted on 28 DN patients (14 early stage and 14 late stage), 12 T2DM patients without any diabetic complications who were referred to the Yazd Diabetes Research and Treatment Center, and 12 healthy people.

Early nephropathy stage (Microalbuminuria) was defined as a urinary albumin level between 30-299 mg/g, and late nephropathy stage (macroalbuminuria) was defined as a urinary albumin level of ≥300 mg/g [20].

The participants in each group were over 30 years old and were well-matched in terms of age and gender.

Patients with the following conditions were excluded from the study: diabetic retinopathy, diabetic neuropathy, type 1 diabetes, secondary diabetes, acute or chronic metabolic or inflammatory diseases, systemic disorders, endocrine disorders, autoimmune diseases, cancers, organ failure, and active infections. These exclusion criteria were implemented to minimize the influence of confounding factors and ensure the homogeneity of the study population.

The demographic information, clinical data, and medical history were obtained with a checklist filled out by the patient’s attending physician.

A volume of 5 mL of whole blood was collected from each study participant and was dispensed into Ethylenediamine tetra-acetic acid (EDTA) blood collection tubes. The blood samples were immediately stored at 4 °C and processed within 2 h of collection to ensure sample integrity. Then, PBMC isolation was performed using the Ficoll solution protocol (Capricorn Scientific, Germany). The isolated PBMCs were resuspended in freezing medium (90% FBS +10% DMSO) and stored at −80 °C for long-term storage until RNA extraction.

### Total RNA extraction and quantitative real-time polymerase chain reaction (qRT-PCR)

Total RNA was extracted from PBMC using the TRIzol reagent (Yekta Tajhiz Azma, Tehran, Iran) according to the manufacturer’s instructions. The extracted RNA was quantified using a NanoDrop™ One spectrophotometer (Thermo Fisher Scientific, MA, United States) and immediately stored at −80 °C in RNase-free tubes until further use. To remove genomic DNA contamination from the RNA samples, a DNase I treatment (Sinaclon, Tehran, Iran) was done according to the manufacturer’s recommendations.

Normalized RNA was then used as a template for reverse transcription and converted into cDNA using a cDNA Reverse Transcription kit (Yekta Tajhiz Azma, Tehran, Iran), following the manufacturer’s protocol. The cDNA samples were stored at −20 °C until qRT-PCR analysis.

Quantitative polymerase chain reaction (q-PCR) was performed to validate the interest genes expression level using the Corbett Rotor-Gene 6000 and Super SYBR Green qPCR master mix (Yekta Tajhiz Azma, Tehran, Iran). Beta-actin (*ACTB*) expression was used as an internal control, and all samples were run in duplicate. Amplification conditions were as follows: 95 °C for 5 min, 40 cycles of 95 °C for 20 s, 60 °C for 20 s, and 72 °C for 30 s. The relative changes in transcript levels were analyzed using the comparative threshold cycle method and ΔΔCT calculations.

All oligonucleotide primers were designed with Primer3.0 plus^3^ and after confirmation of target-specific primers by Primer-BLAST^4^, were purchased from Metabion International AG (Planegg, Germany). The primers were reconstituted in nuclease-free water, aliquoted, and stored at −20 °C to prevent degradation.

The primer sequences used were the following:

*MCF2L-AS1* (Gene ID: 100289410) (forward 5′CGC​AGC​TAT​CCT​TTT​GTG​GT3′,
reverse 5′AAC​TGA​TTG​GGG​AGT​GAG​GT3′),
BCOR (Gene ID: 54880) (forward 5′AAA​GTC​GGT​CAC​CCT​GGA​G3′,
reverse 5′CTT​CAA​AGG​GAT​CAC​GGT​GC3′),
*ACTB* (Gene ID: 60) (forward 5′GCC​TCG​CCT​TTG​CCG​AT3′,
reverse 5′TTC​TGA​CCC​ATG​CCC​ACC​AT3′).

### Statistical analysis

Statistical analyses were implemented with the GraphPad Prism 8 software.

Continuous variables with normal distribution were shown as the mean ± standard deviation (SD). Categorical variables were represented as frequency (percentage) and analyzed by a chi-squared test. The normality of the data was assessed using the Shapiro-Wilk test. For datasets that exhibited normal distributions, we employed one-way ANOVA followed by *post hoc* Tukey’s test. In contrast, the Kruskal-Wallis test was utilized for datasets that did not meet the assumptions of normality. Spearman correlation was used to analyze the correlation between mRNA gene expression level and clinicopathological parameters.

The receiver operating characteristic (ROC) curve was drawn to determine the predictive value of lncRNA for the diagnosis. A p-value <0.05 was considered statistically significant.

## Results

### Identification of DEGs

A microarray gene expression dataset (GSE142153) was utilized for this investigation. The analysis focused on PBMCs obtained from individuals with varying degrees of diabetic nephropathy, utilizing microarray technology. The dataset consists of three different experimental conditions, namely 10 healthy control samples, 23 samples of diabetic nephropathy, and 7 samples of ESRD, totaling 40 samples. The data was subjected to analysis using the limma package, resulting in the identification of 372 DEGs, with 147 exhibiting up-regulation and 225 displaying down-regulation. All DEGs were identified between the diabetic (n = 23) and normal (n = 10) samples. Among the identified differentially expressed genes, the long non-coding RNA *MCF2L-AS1* was selected for further investigation due to its potential functional relevance. Subsequently, we focused on characterizing the predicted target genes of *MCF2L-AS1* to explore its possible regulatory roles in diabetic nephropathy.

### Finding targets for each *MCF2L-AS1*

**FIGURE 1:**
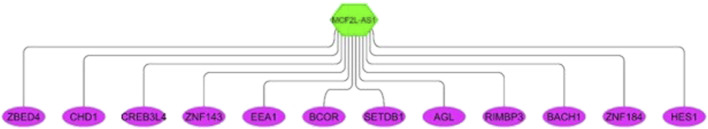
Networks were constructed between *MCF2L-AS1* and targets.

By RNAInter, we determined 540 proteins as targets for *MCF2L-AS1*. After confirming the obtained targets from RNAInter and DEmRNAs, *MCF2L-AS1* and 12 target interactions were finally constructed using Cytoscape software (Table 1; Figure 1).

**TABLE 1:** Final targets regulated by *MCF2L-AS1*.

No.	Symbol	Full name
1	EEA1	Early endosome antigen 1
2	CREB3L4	cAMP responsive element binding protein 3 like 4
3	ZBED4	Zinc finger BED-type containing 4
4	ZNF184	Zinc finger protein 184
5	RIMBP3	RIMS binding protein 3
6	AGL	Amylo-alpha-1, 6-glucosidase, 4-alpha-glucanotransferase
7	SETDB1	SET domain bifurcated histone lysine methyltransferase 1
8	BCOR	BCL6 corepressor
9	ZNF143	Zinc finger protein 143
10	CHD1	Chromodomain helicase DNA binding protein 1
11	HES1	hes family bHLH transcription factor 1
12	BACH1	BTB domain and CNC homolog 1

### GO analysis

**FIGURE 2:**
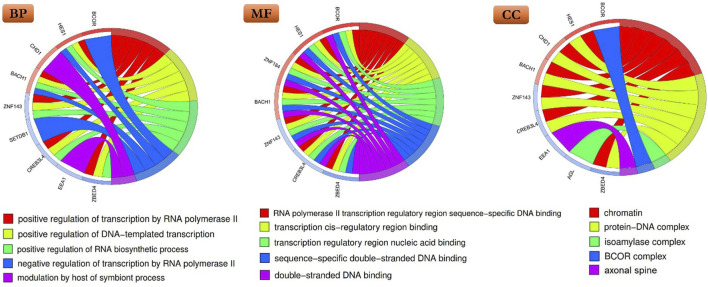
Chord plots illustrating the top five enriched gene ontology (GO) terms for the Biological Process (BP), Molecular Function (MF), and Cellular Component (CC) of final targets of *MCF2L-AS1*.

No significantly enriched canonical biological pathways were identified in our study. In the GO enrichment analysis, the top five enriched terms from each of the three GO categories -BP, MF, and CC- were selected. This approach provides a comprehensive overview and avoids potential bias towards any single category. For the BP category, included terms were positive regulation of transcription by RNA polymerase II, positive regulation of DNA-templated transcription, positive regulation of RNA biosynthetic process, negative regulation of transcription by RNA polymerase II, and modulation by host of symbiont process. In the MF category, the most relevant terms identified were RNA polymerase II transcription regulatory region sequence-specific DNA binding, transcription cis-regulatory region binding, transcription regulatory region nucleic acid binding, sequence-specific double-stranded DNA binding, and double-stranded DNA binding. In the CC category, enriched terms encompassed chromatin, protein-DNA complex, isoamylase complex, BCOR complex, and axonal spine (Figure 2). The complete list of enriched GO terms is provided in Supplementary Material S1.

### Demographic and clinical data evaluation

As shown in Table 2, there was no significant difference in terms of age (P = 0.069), gender (P = 0.759), TG (mg/dL) (P = 0.172), and HDL (mg/dL) (P = 0.619) between the groups.

**TABLE 2:** Demographic and laboratory characteristics of participants.

Variable	Non-diabetic	T2DM	Early DN	Late DN	P-value
Male	8 (66.66%)	10 (62.5%)	12 (75%)	11 (78.57%)	0.759*
Female	4 (33.33%)	6 (37.5%)	4 (25%)	3 (21.42%)	​
Age (year)	53.67 ± 10.72	57.83 ± 9.00	63.23 ± 7.80	60.43 ± 11.80	0.069**
Hb A1c (%)	5.53 ± 0.29	7.4 ± 1.64	7.657 ± 0.98	7.76 ± 1.7	0.002***
TG (mg/dL)	251.5 ± 86.44	185.4 ± 82.53	175.8 ± 77.79	167.2 ± 99.41	0.172**
Chol (mg/dL)	215.6 ± 23.66	183.4 ± 34.77	158.9 ± 37.33	156.1 ± 27.15	<0.001***
HDL (mg/dL)	40.75 ± 5.72	46.33 ± 10.5	40.25 ± 11.01	44.27 ± 13.01	0.619***
LDL (mg/dL)	124.6 ± 28.38	94.87 ± 33.93	80.78 ± 33.58	84.10 ± 26.87	0.021***
Cr (mg/dL)	0.82 ± 0.10	0.83 ± 0.11	1.07 ± 0.33	2.13 ± 1.11	<0.001**
GFR (mL/min/1.73m^2^)	99.19 ± 14.24	90.90 ± 7.89	78.54 ± 21.18	45.71 ± 30.58	0.001**
Albumin (mg/g)	7.60 ± 4.50	14.13 ± 2.16	123.9 ± 71.67	795.4 ± 555.00	<0.001**

Hb A1c, glycated hemoglobin; TG, triglyceride; Chol, cholesterol; HDL, high-density lipoprotein; LDL, low-density lipoprotein; Cr, creatinine; GFR, glomerular filtration rate. Data presented as mean ± standard deviation (SD). A p-value <0.05 was considered significant.

*Chi-square test was used.

**Kruskal-Wallis test was used.

***One-way ANOVA test was used.

### Expression of *MCF2L-AS1*and *BCOR* gene in the studied groups

**FIGURE 3:**
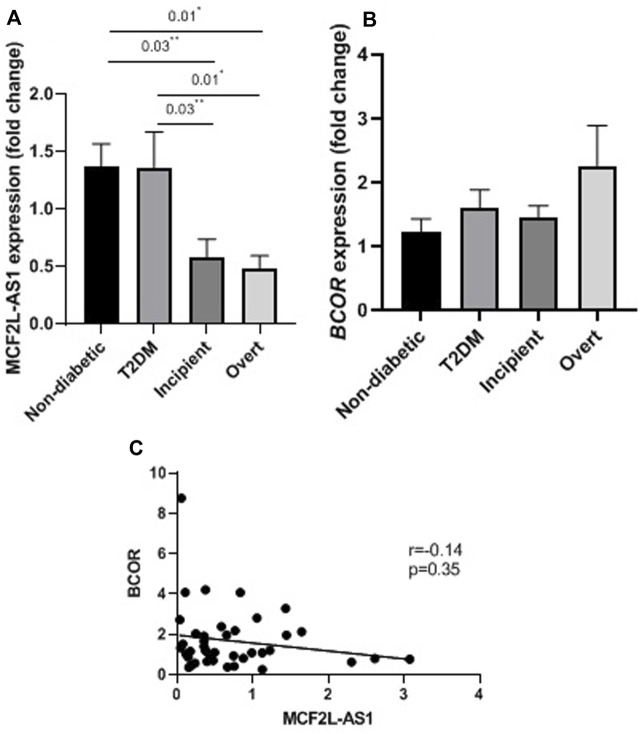
Expression levels of *MCF2L_AS1* and *BCOR* and their correlation in the studied groups. **(A)** Relative expression of *MCF2L_AS1*. **(B)** Relative expression of *BCOR*. **(C)** Correlation between *MCF2L_AS1* and *BCOR* expression levels. Statistical power for the comparisons was 81% (**) and 88% (*), respectively.

In this study, the expression level of the *MCF2L-AS1* and *BCOR* gene in different stages of DN was compared to T2DM and non-diabetic groups by the qRT-PCR method. Tukey’s test analysis showed that the expression of *MCF2L-AS1* decreased in the early DN group compared to the T2DM and non-diabetic group (P = 0.01). Furthermore, the expression of this lncRNA declined in the late DN group compared to the T2DM and non-diabetic group (P = 0.03). However, the *MCF2L-AS1* expression level was not significantly different between the different stages of DN, including the early and over groups (P = 0.98) (Figure 3A).

Moreover, *BCOR* gene expression analysis revealed no significant difference between the groups (P = 0.27) (Figure 3B). Moreover, the correlation between the expression levels of *MCF2L-AS1* with *BCOR* was not significant (p = 0.35, r = −0.14, CI = −0.44 to 0.17) (Figure 3C).

### Clinicopathological correlation analysis of *MCF2L-AS1*and *BCOR*

**FIGURE 4:**
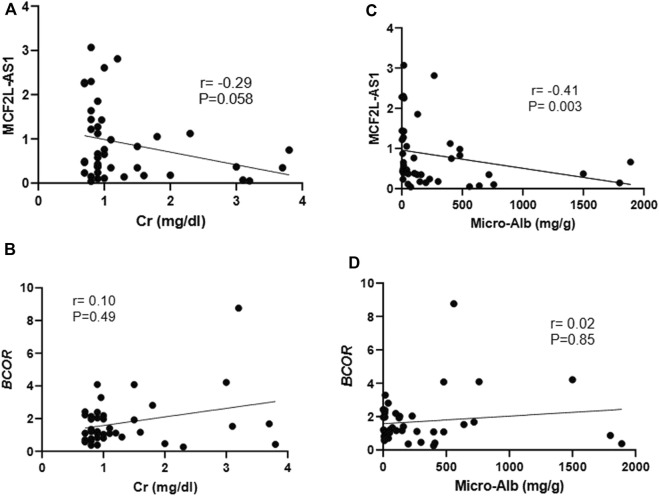
Correlation analysis of Cr (mg/dl) with *MCF2L-AS1* **(A)** and *BCOR* **(B)** gene expressions. and the correlation analysis of microalbumin (mg/g) with *MCF2L-AS1* **(C)** and *BCOR* **(D)** gene expressions.

As the expression of *MCF2L-AS1* was significantly different between the DN and control groups, we further investigated the correlation between expression levels and clinicopathological parameters. The results demonstrated that Cr (mg/dL) had no relationship with the expression level of *MCF2L-AS1* (P = 0.058, r = −0.29, CI = −0.55 to 0.02) (Figure 4A) and a negative relationship with microalbumin (mg/g) (P = 0.003, r = −0.41, CI = −0.64 to −0.11) (Figure 4C). In contrast, there was no relationship between the expression level of *BCOR* and Cr (mg/dL) (P = 0.49, r = 0.10, CI = −0.21–0.41) (Figure 4B) and microalbumin (mg/g) (P = 0.85, r = 0.02, CI = −0.28–0.34) (Figure 4D).

### Evaluation of the specificity and sensitivity of *MCF2L-AS1* as a diagnostic biomarker of DN

**FIGURE 5:**
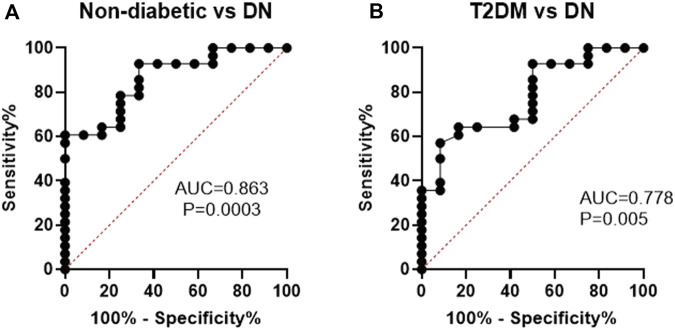
Evaluation of the ability of *MCF2L-AS1* to diagnose individuals with DN from non-diabetic **(A)** and *T2DM* **(B)** individuals.

To assess the diagnostic value of *MCF2L-AS1* as a biomarker for DN, ROC curve analysis was performed. As shown in Figure 5A, *MCF2L-AS1* could differentiate significantly between non-diabetic and DN groups as an independent variable, with an area under the ROC curve (AUC) of 0.863, using a threshold of <0.850, a sensitivity of 78.57%, and a specificity of 75%. Furthermore, the AUC yielded 0.778, with a sensitivity of 64.29% and a specificity of 83.33% (threshold of <0.480) to distinguish between the T2DM and DN groups (Figure 5B).

## Discussion

Understanding the molecular mechanisms and the specific genes involved in the pathogenesis of DN is crucial for devising effective management strategies [21]. Such insights not only enhance our comprehension of the disease’s mechanisms but also facilitate the development of targeted therapeutic interventions [7].

Long noncoding RNA *MCF2L-AS1* serves as a newly found lncRNA, and studies have suggested its role as an oncogene in various cancers, especially colorectal and lung cancers [19, 22]. Although studies have discovered the role of *MCF2L-AS1* in metastasis, proliferation, invasion, and migration [17, 18] its role in DN remains poorly understood.

Based on the best of our knowledge, there is no study regarding the role of *MCF2L-AS1* in DN. In the present study, for the first time, we demonstrated that the expression of *MCF2L-AS1* was markedly decreased in the PBMCs of individuals with DN compared to those with T2DM and non-diabetic groups.

DN is characterized by a state of systemic inflammation and immune dysregulation [23]. This pathological situation leads to the activation of PBMCs [24], which subsequently infiltrate the renal tissue [25]. Within the kidney, these activated immune cells contribute directly to parenchymal injury by releasing pro-inflammatory cytokines and pro-fibrotic factors [25, 26]. The activated state of these cells is reflected in their molecular signature and transcriptome, which includes specific alterations in lncRNA expression profiles such as *MCF2L-AS1* [27]. Consequently, measuring *MCF2L-AS1* levels in circulating PBMCs (as an accessible sample) provides a minimally invasive window into the underlying inflammatory processes driving renal pathology.

To determine whether *MCF2L-AS1* was associated with the severity and prognosis of DN, the correlation between *MCF2L-AS1* levels and clinicopathological parameters of DN, including Cr (mg/dL) and microalbumin (mg/g) levels, was further evaluated. Our results showed a negative correlation between *MCF2L-AS1* expression level and microalbuminuria (mg/g). These significant correlations with markers of renal function provide strong circumstantial evidence that *MCF2L-AS1* expression in PBMCs is tied to the severity of kidney damage.

The absence of significant differences in *MCF2L-AS1* expression between the early and late DN groups proposes that the primary dysregulation of *MCF2L-AS1* occurs during the initial phases of the disease. While these findings indicate an association, they do not support a sustained, strong influence for this lncRNA across all disease stages.

On the other side, the ROC curve analysis demonstrated that *MCF2L-AS1* has significant predictive value in distinguishing DN patients from both T2DM patients without complications and non-diabetic individuals.

In addition, the GO enrichment analysis of *MCF2L-AS1* target genes revealed a significant overrepresentation of terms related to transcriptional regulation, like “positive regulation of transcription by RNA polymerase II” and chromatin organization, like “chromatin,” *“BCOR* complex” (Figure 2). This allows us to hypothesize that the potential role of *MCF2L-AS1* in DN may be mediated through epigenetic mechanisms and the control of gene expression. However, correlation analysis between *MCF2L-AS1* and *BCOR* expression did not reveal a significant association. This lack of correlation might be explained by the fact that *BCOR* was assessed only at the mRNA level. Therefore, it is recommended that *BCOR* expression be investigated at the protein level in future studies.

These findings suggest that *MCF2L-AS1* may be a disease-associated molecule in diabetic nephropathy, particularly in the early stages of the disease. However, given the current study design and data, definitive conclusions regarding its diagnostic utility or central mechanistic role cannot be made. Further studies with larger cohorts and refined patient stratification are required to elucidate its potential as a biomarker and its involvement in disease mechanisms.

In several studies, it has been revealed that *miR-874-3p* is a target of *MCF2L-AS1*, and *MCF2L-AS1* sponges *miR-874-3p* in colorectal cancer [17, 22]. Sham et al. found that circulating *miR-874-3p* is upregulated in the sera of individuals with T2DM and macroalbuminuria. Additionally, they reported a negative correlation between eGFR and *miR-874-3p* [28]. Another study by Zhang et al. demonstrated that *MCF2L-AS1* suppresses the expression of *miR-874-3p*, leading to the upregulation of *FOXM1* in colorectal cancer [17]. *FOXM1* has a protective role against renal damage and alleviates podocyte pyroptosis in patients with DN [29]. Several studies indicated that *FOXM1* expression declined after kidney damage [29–31].

As a result, based on established mechanisms in other pathologies, we hypothesize that the observed downregulation of *MCF2L-AS1* in our DN group might lead to an increase in *miR-874-3p* activity, potentially resulting in the suppression of its target, *FOXM1*, thereby exacerbating renal damage. This proposed mechanism, bridging a lncRNA from oncogenic research to diabetic complications, underscores the utility of exploring conserved molecular networks and provides a compelling mechanistic hypothesis for the role of *MCF2L-AS1* in DN. Accordingly, while our data do not provide functional validation, it could be one speculative mechanism worthy of future investigation by measuring *miR-874-3p* and *FOXM1* expression in DN patients.

On the other side, it has been reported that silencing of *MCF2L-AS1* increases the expression of *miRNA-33a* [32]. The upregulation of *miRNA-33* has been observed in the serum of DN patients and in the sera and renal tissues of DN model rats [33]. Liu et al. indicated that sponging *miR-33a-5p* by circ-ITCH leads to alleviating renal inflammation and fibrosis in diabetic model rats [34]. As a result, it is suggested that further investigation be conducted into the downstream effects of altered *MCF2L-AS1* expression on key molecular players in DN pathways.

This study had several limitations. Although we used the microarray gene expression dataset (GSE142153) obtained from PBMCs of patients with diabetic nephropathy, it should be noted that PBMCs may not fully capture the complex, renal-specific molecular mechanisms underlying diabetic nephropathy. Indeed, a key limitation of this study is the lack of direct investigation of *MCF2L-AS1* expression in kidney tissue. So, future studies directly measuring *MCF2L-AS1* in kidney tissue are essential to establish a direct link between its circulating levels and its activity within the renal environment and to confirm its utility as a systemic biomarker.

Secondly, the lack of functional assays, such as gain or loss-of-function studies, impedes a direct demonstration of *MCF2L-AS1’s* role in DN pathogenesis. Thirdly, the relatively small sample size necessitates validation in larger, multi-center cohorts to strengthen the robustness of our findings. Furthermore, while bioinformatics predicted *BCOR* as a target, the absence of a significant correlation in their expression levels suggests a more complex relationship that may involve indirect mechanisms not captured at the mRNA level. Finally, the study groups lacked finer stratification, such as patients with normoalbuminuric diabetic kidney disease, which could offer additional insights into early molecular changes.

Addressing these limitations in future research through experimental validations and comprehensive pathway analyses will clarify the precise molecular interactions and enhance understanding of the clinical utility of molecular biomarkers in DN.

## Conclusions

In conclusion, our findings introduce *MCF2L-AS1* as a novel, promising, disease-associated molecule worthy of further investigation as a potential non-invasive biomarker for DN, rather than a confirmed diagnostic tool. The declined expression in individuals with DN compared to T2DM and non-diabetic groups and an observed correlation with microalbumin, suggest a multifaceted role for *MCF2L-AS1* in the pathogenesis of DN. This research contributes to a deeper comprehension of the molecular mechanisms underlying DN and opens avenues for further exploration.

## Data Availability

The original contributions presented in the study are included in the article/Supplementary Material, further inquiries can be directed to the corresponding author.

## Footnotes

1
http://www.ncbi.nlm.nih.gov/geo

2
https://bioinfogp.cnb.csic.es/tools/venny/

3
http://bioinfo.ut.ee/primer3-0.4.0/

4
http://www.ncbi.nlm.nih.gov/tools/primer-blast

## Supplementary material

Supplementary Table S1 The complete results of the Gene Ontology enrichment analysis.
